# Clinical Neuropathology image 4-2017: High-resolution 7 Tesla MRI of postmortem brain specimens: improving neuroimaging-neuropathology correlations 

**DOI:** 10.5414/NP301049

**Published:** 2017-06-28

**Authors:** Mar Guasp-Verdaguer, Oriol Grau-Rivera, Alberto Prats-Galino, Núria Bargalló, Raquel Sánchez-Valle, Ellen Gelpi, Guadalupe Soria

**Affiliations:** 1 Neurology Department, Hospital Clínic de Barcelona,; 2 Laboratory of Surgical NeuroAnatomy, Human Anatomy and Embryology Unit, Faculty of Medicine, Universitat de Barcelona,; 3 Radiology Department, Hospital Clínic de Barcelona,; 4 Magnetic Resonance Image Core Facility, Institut d’Investigacions Biomèdiques August Pi I Sunyer (IDIBAPS), Barcelona, and; 5 Neurological Tissue Bank of the Hospital Clinic-IDIBAPS, Barcelona, Spain

**Keywords:** high-resolution MRI, 7T, postmortem brain imaging

## Abstract

No Abstract available.

The application of high-resolution ultra-high field MRI or 7 Tesla (7T) MRI on postmortem, formalin-fixed brain tissue allows direct comparison of neuroimaging with detailed neuropathological analyses. This technique has been mainly used in the field of multiple sclerosis [1, 2, 3, 4, 5, 6] but also for the study of white matter tracts [7, 8]. Some authors have observed differences in diffusion parameters between in vivo and postmortem imaging, as well as before and after formalin fixation, likely caused by cell lysis and chemical change of molecules, factors that may limit the interpretation of results. 

Recently, Stefanits et al have analyzed the feasibility of in vivo 7T MRI in the clinical setting in patients with temporal lobe epilepsy [9]. The authors observed a strong correlation between imaging findings and histology, specifically between volume loss and signal intensity on MRI, and overall grading of neuronal loss and astrogliosis of the hippocampal subfields CA1-4 on histology after surgical removal. The major limitations were the long acquisition times to achieve high-resolution images and the presence of artifacts. 

Despite these limitations, high-resolution ultra-high-field MRI is a promising tool for the detection of subtle histopathological changes in vivo. It might be also applied postmortem to analyze the fine structure and circuits of the human brain as well as to several neurological conditions including neurodegenerative diseases, in order to find better anatomical correlates and improve diagnostic markers of disease. 

## Conflict of interest 

The authors report no conflict of interest. 

**Figure 1.**
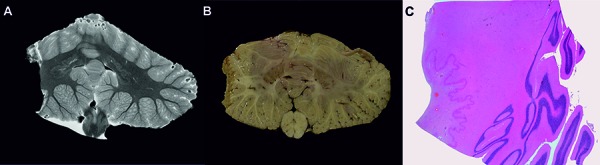
A: 7T MRI of cerebellar cortex nicely depicts the 3 layers of the cortex and the zick-zack profile of the dentate nucleus; B: formalin-fixed cerebellum of the same patient, C: H & E-stained section of the dentate nucleus.
